# The Vav GEF Family: An Evolutionary and Functional Perspective

**DOI:** 10.3390/cells8050465

**Published:** 2019-05-16

**Authors:** Sonia Rodríguez-Fdez, Xosé R. Bustelo

**Affiliations:** 1 Centro de Investigación del Cáncer, Consejo Superior de Investigaciones Científicas (CSIC) and University of Salamanca, Campus Unamuno, E37007 Salamanca, Spain; soniarf@usal.es; 2 Instituto de Biología Molecular y Celular del Cáncer, Consejo Superior de Investigaciones Científicas (CSIC) and University of Salamanca, Campus Unamuno, E37007 Salamanca, Spain; 3 Centro de Investigación Biomédica en Red de Cáncer (CIBERONC), Consejo Superior de Investigaciones Científicas (CSIC) and University of Salamanca, Campus Unamuno, E37007 Salamanca, Spain

**Keywords:** Rho GTPases, Dbl-homology, guanosine diphosphate to guanosine triphosphate exchange factors, protein tyrosine kinases, signaling, evolution, phosphorylation, tyrosine, animal models

## Abstract

Vav proteins play roles as guanosine nucleotide exchange factors for Rho GTPases and signaling adaptors downstream of protein tyrosine kinases. The recent sequencing of the genomes of many species has revealed that this protein family originated in choanozoans, a group of unicellular organisms from which animal metazoans are believed to have originated from. Since then, the Vav family underwent expansions and reductions in its members during the evolutionary transitions that originated the agnates, chondrichthyes, some teleost fish, and some neoaves. Exotic members of the family harboring atypical structural domains can be also found in some invertebrate species. In this review, we will provide a phylogenetic perspective of the evolution of the Vav family. We will also pay attention to the structure, signaling properties, regulatory layers, and functions of Vav proteins in both invertebrate and vertebrate species.

## 1. Introduction

Guanosine nucleotide exchange factors (GEFs) are enzymes that catalyze the exchange of guanosine diphosphate (GDP) by guanosine triphosphate (GTP) in Rho proteins, thereby allowing the rapid transition of those GTPases from the inactive (GDP-bound) to the active, GTP-bound state during cell signaling. Given their multidomain structure, GEFs contribute to fine-tune the activation of Rho GTPases both in time and space in cells. In addition, they can participate in some cases in the predetermination of the effector-binding spectra of the GTPases as well as in signaling diversification events via the engagement of GTPase-independent pathways [1,2]. To accommodate all these tissue- and cell type-specific regulatory requirements, the Rho GEF family has become highly diversified during evolution. In mammals, for example, there are more than 80 different Rho GEFs [1,3]. Depending on the type of catalytic domain, the Rho GEFs can be ascribed to either the Dbl-homology (DH) or the dedicator of cytokinesis (Dock) subfamilies [1].

The Vav family is one of the best-known groups of DH containing GEFs [4]. These proteins behave as phosphorylation-dependent molecular switchers, fluctuating between an inactive (nonphosphorylated) and active (tyrosine phosphorylated) conformation during signal transduction. Vav proteins play critical roles in many biological processes controlled by protein tyrosine kinases (PTKs) both in invertebrate and vertebrate species. They also epitomize the regulatory complexity of most Rho GEFs, since they can also act as adaptor proteins [4]. Recent results have shown that Vav proteins are critical for the homeostasis of the central nervous, cardiovascular, and immune systems [4]. They also contribute to several pathologies such as cancer and immune system-related pathologies [3,4]. As a result, they are currently considered potential therapeutic targets for several pathological conditions [4].

Vav1 (formerly known as Vav and p95^vav^) was the first member of the Vav family ever identified during the search for new oncogenes in the late 1980s of the past century [5]. Since it was the sixth human oncogene identified by the Barbacid’s group, this new locus was designated as the sixth letter of the Hebrew alphabet (*vav*). The two other Vav family proteins present in mammals, Vav2 and Vav3, were discovered during the 1995–1999 period using standard cloning procedures [6,7,8]. Since then, many members of the Vav family have been found in other species. This has made it possible, for the first time, to get a holistic view of the evolution of this GEF family. As it will be seen in this review, we now know that Vav proteins originated at the very transition between unicellular and multicellular organisms. Although the “body plan” of these proteins shows high phylogenetic conservation, we have found events associated with the loss of specific structural domains, changes in the spatial arrangement of those domains within the overall protein, and variations in the number of Vav family members that are associated with specific evolutionary steps. To facilitate the understanding of these evolutionary changes, we will also include in this review information on the structure, the regulation, and the most important biological functions of these proteins. Information on Vav proteins not covered in this work can be found in previous review articles [4,9].

## 2. Structure

**Figure 1:**
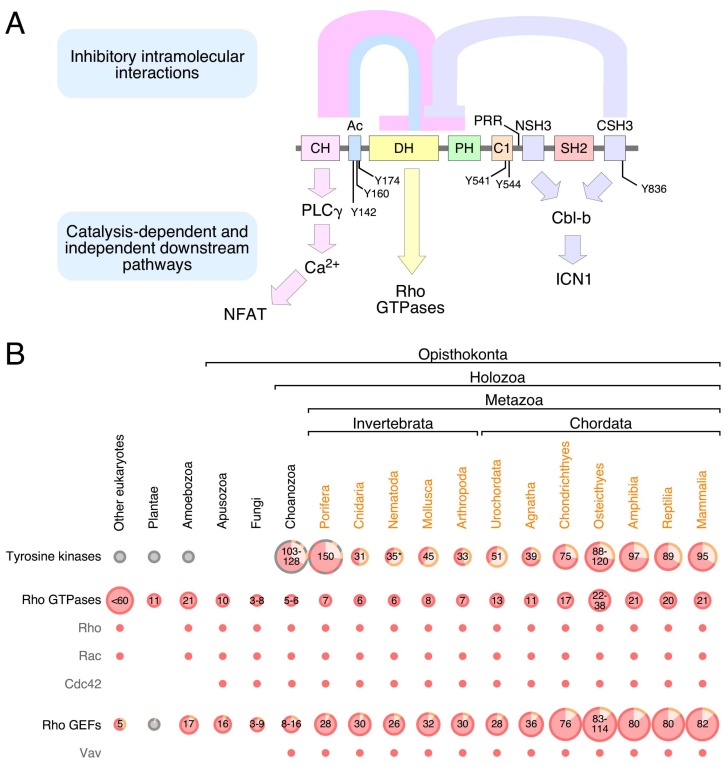
Structure, regulation, effector functions, and evolution of Vav proteins. (**A**) Depiction of the structure, the intramolecular interactions that control the signaling output (top), the main regulatory phosphosites (using the amino acid sequence corresponding to mouse Vav1), and the main downstream pathways (bottom) of mammalian Vav proteins. Abbreviations for the domains and proteins depicted in the figures have been described in the main text. (**B**) Evolution of PTKs, Rho GTPases, and Rho GEFs. The size of the circles is proportional to the number of members present in the indicated protein families and taxonomic groups. Please note that in some species (e.g., amoebozoans, plants) there is tyrosine kinase activity carried out nonspecifically by serine/threonine protein kinases (gray circles). Proteins with equivalent function but with non-metazoan domain combinations are also present in other species (gray circles). In the case of PTKs, the percentage of transmembrane (light red color) and cytoplasmic (light brown color) versions is depicted in the figure. For Rho GEFs, the percentage of members belonging to the DH and Dock subclasses is indicated using light red and light brown colors, respectively. In the case of Rho GTPases and Rho GEFs, the presence of specific family members (Rho, Rac, Cdc42, Vav) is indicated by small light red dots. This figure integrates information from previous publications [19,20,21,22,23,24,25,26] and manual curation procedures using UniProt-archived data.

Mammalian Vav family proteins contain a common structural scaffold composed of an N-terminal calponin-homology (CH) domain, an acidic (Ac) region, the catalytic DH domain, a pleckstrin-homology (PH) region, a C1 subtype zinc finger (ZF) domain, a proline rich region (PRR), and a SH3-SH2-SH3 cassette (Figure 1A). Some of these regions adopt more complex superorder structures in the context of the entire molecule. Thus, the DH, PH, and ZF domains form a common enzymatic core that is essential for full catalytic competency [10,11,12]. This DH-PH-ZF cassette is quite unique, since it is not present in the rest of known Rho GEFs. This catalytic cassette can establish further interactions with both the CH-Ac and the most C-terminal SH3 (CSH3) regions depending on the phosphorylation state of the protein [8,10,13,14,15] (Figure 1A). Finally, the Vav PRR and the most N-terminal SH3 (NSH3) domain form a single structural unit at least in the case of Vav1. This unit facilitates the interaction of specific protein partners such as Grb2 [16,17]. Vav proteins are the only Ras superfamily GEFs that harbor a SH2 region (Figure 1A), the phosphotyrosine binding domain that is commonly used to mediate interactions with either autophosphorylated PTKs or transphosphorylated signaling mediators [18]. This feature further highlights the close connection between Vav proteins and PTK-regulated pathways.

## 3. Signaling Functions

Vav proteins mainly act as enzymes that catalyze the activation step of Rho subfamily GTPases during cell signaling [8,14,27,28,29] (Figure 1A). As indicated above, this function is mediated by a multidomain cassette composed of the catalytic DH domain and the adjacent PH and ZF regions [11,12]. However, Vav proteins can also play adaptor-like functions in specific signaling contexts. The oldest known of these catalysis-independent functions is the stimulation of the nuclear factor of activated T cells (NFAT) in lymphocytes (Figure 1A). Although not fully dissected at the mechanistic level as yet, it is known that this pathway requires the CH domain of Vav proteins, the downstream stimulation of phospholipase Cγ (PLCγ), the PLCγ-mediated production of inositol 1,4,5-triphosphate (IP_3_), the IP_3_-dependent generation of Ca^2+^, the Ca^2+^-dependent stimulation of the calcineurin phosphatase, and the ensuing dephosphorylation and nuclear translocation of NFAT. The full activation of this pathway also requires the engagement of synergistic signals generated by the stimulation of the upstream T cell receptor [15,30,31,32,33,34,35]. The CH domain of Vav proteins is also involved in the binding to the methyltransferase EZH2. This binding seems to be important for the formation of cytosolic signaling complexes involved in talin 1 methylation, cell adhesion, and tumorigenesis. However, it is unclear as yet whether this function is catalysis—dependent or independent [36]. The most recent adaptor-like function of these proteins is the negative regulation of the intracellular active fragment of Notch1 (ICN1) by Vav1 (Figure 1A). This function, which is mediated by the Vav1 SH3 domains, favors the formation of cytosolic complexes between ICN1 and the E3 ubiquitin ligase Cbl-B (Cas-Br-murine ecotropic retroviral transforming sequence 2) that, in turn, facilitates the Cbl-B-mediated ubiquitinylation of ICN1 and the ensuing degradation of the Notch1 fragment in the proteasome. This tumor suppressor-like function is highly relevant for T cell leukemogenesis. For example, we have recently shown that the elimination of the *Vav1* gene in mice results in increased ICN1 abundance in immature T cells, exacerbated ICN1 signaling, and the long-term development of Notch1-dependent immature T cell acute lymphoblastic leukemia [37,38,39]. Conversely, the restoration of this pathway in patient-derived T cell acute lymphoblastic leukemia cells leads to impaired tumorigenesis [37,38,39]. It is likely that other GTPase-independent pathways are stimulated by Vav proteins in other cell types. For example, the analysis of the Vav-dependent transcriptome of breast cancer cells using genome expression profiling experiments has revealed that some of the Vav2-dependent genes can be also regulated when using a catalytically-dead Vav2 protein [40]. Whereas most of the foregoing catalysis-independent roles take place in the cytosol, there are also data supporting the participation of Vav1 and Vav3 in adaptor-like functions in the nucleus of both hematopoietic and nonhematopoietic cells. These functions require the CSH3 domain and the DH-PH-ZF cassette of Vav1 and Vav3, respectively [41,42,43,44,45].

Vav proteins also contribute to signaling diversification downstream of membrane receptors by promoting the production of key second messengers using either catalysis-dependent or independent mechanisms. The former ones include the Vav2-mediated production of cGMP in nitric oxide-stimulated vascular smooth muscle cells and the Vav2- and PLCγ-dependent generation of diacylglycerol and IP_3_ in stimulated B lymphocytes. The catalysis-independent pathways include the CH-mediated stimulation of diacylglycerol and IP_3_ production in T cells described above [33,46,47,48]. These second messengers allow the activation of pathways that are not typically stimulated by Rho GEFs and Rho proteins such as, for example, the diacylglycerol-mediated stimulation of the Ras GDP releasing factor 1 (Ras GRF1)-Ras-Raf-Erk axis in both T- and B-cells [34,47,49,50,51,52].

Despite sharing the same structure, it is important to note that current evidence indicates that Vav proteins display overlapping but not identical functions. Consistent with this, it has been shown that Vav1 and Vav3, but not Vav2, can promote the stimulation of NFAT in T lymphocytes [13,53]. Vav1 is also the main family member involved in the negative regulation of the Notch1 pathway in T cells [37]. Genome expression profiling experiments further indicate that Vav2 and Vav3 promote both common and family member-specific changes in the transcriptome of breast cancer cells [40]. As a result, only the expression of both proteins can rescue the defects in primary tumorigenesis and lung metastasis that are exhibited by cancer cells lacking both Vav2 and Vav3 [40]. By contrast, there are specific functions associated with the maintenance of breast cancer epithelial integrity that can be redundantly performed by Vav2 and Vav3 [54].

## 4. Regulation

The biological activity of Vav proteins is controlled by phosphorylation-dependent conformational changes. The nonphosphorylated Vav proteins are inactive due to a “closed” conformation mediated by extensive intramolecular interactions between the CH-Ac and the CSH3 with the catalytic DH-PH-ZF core (Figure 1A). These interdomain interactions occlude the GTPase binding site and, at the same time, favor a conformation of the DH-PH-ZF cassette that is not compatible with full catalytic efficiency [8,10,13,14,15,55]. Many adaptor-like functions of Vav proteins are also subjected to this type of regulation, indicating that those interdomain interactions also contribute to the occlusion of noncatalytic effector sites of the molecule. By contrast, the suppressor activity of Vav1 that contributes to the buffering of Notch1 signaling can be fully engaged by both the phosphorylated and nonphosphorylated versions of the protein [37]. In stimulated cells, Vav proteins acquire an “open”, biologically active state upon becoming phosphorylated on several tyrosine residues located in the Ac (Tyr^142^, Tyr^160^, Tyr^174^), ZF (Tyr^541^, Tyr^544^), and CSH3 (Tyr^836^) domains (please note that the amino acid numbers are given considering those present in the primary sequence of mouse Vav1) [8,10,13,14,15,55,56] (Figure 1A). It is not currently known whether the CH-Ac and CSH3 regions fully detach from the rest of the domains of the molecule during this activation step or whether they remain in contact with other domains using a different spatial orientation. However, functional data obtained using several Vav1 point mutants suggest that the CH-Ac region has to remain bound to other parts of the molecule in order to promote the optimal stimulation of the NFAT pathway in T lymphocytes [57]. In line with this, electron microscopy experiments also suggest that the Vav1 CSH3 maintains interdomain contacts with the catalytic core in the “open” conformation of the protein [13]. It is likely that the foregoing conformational changes are used in additional regulatory steps. For example, the recent use of phosphosite mutant proteins revealed that Vav1 can engage different downstream signaling branches depending on the total number of phosphosites that are phosphorylated at a given time [57]. This suggests that Vav proteins can fluctuate between different conformational and signaling states during cell signaling.

The phosphorylation step of Vav proteins is mediated by transmembrane and/or cytosolic PTKs depending on the cell type involved. In the former case, the activation step involves the direct, SH2-mediated interaction of Vav proteins with the autophosphorylated cytoplasmic tail of the upstream PTK [58,59]. In the latter case, the process is much more complex since it requires the prior translocation of Vav proteins to the plasma membrane via interactions with either transmembrane coreceptors (i.e., CD19) or cytosolic adaptor molecules (e.g., Slp76, Blnk, Grb2, Nck, or dynamin 2). These interactions are mediated by the SH2- (CD19, Slp76, Blnk), the PRR-NSH3- (Grb2, Nck) or the CSH3 (dynamin 2) of Vav proteins [53,60,61,62,63,64,65,66,67,68,69,70,71]. Adding further complexity to this activation step, recent experiments have shown that the final phosphorylation step in this case requires multiple and concurrent inputs from several cytoplasmic PTKs. Consistent with this, the optimal phosphorylation kinetics of Vav1 downstream of the T cell receptor requires the independent engagement of the Lck and Zap70 PTKs. These kinases also have different roles in the phosphorylation of specific tyrosine residues of Vav1 (e.g., the Y^836^ phosphosite) [57]. Interestingly, it has also been found that two Vav1 downstream elements, Slp76 and PLCγ1, are involved in a negative feedback mechanism that contributes to maintain low levels of Vav1 tyrosine phosphorylation in nonstimulated T cells. As a result, high levels of basal Vav1 phosphorylation are detected in naïve T cells lacking either Slp76 or PLCγ1 [57]. The signaling mechanism that mediates this feedback loop is currently unknown, although current data indicate that it does not require the catalytic activity of PLCγ1 [57].

The relevance of this intramolecular mechanism is underscored by the gain-of-function effects observed when the specific domains and regulatory sites of Vav proteins involved in the maintenance of the “closed” conformational state are either removed or inactivated by point mutations [7,8,10,13,14,15,48,56,72]. Perhaps more importantly, mutations in the *VAV1* gene that cause the elimination of those intramolecular interactions have also been found in human tumors [9,38,73,74,75,76,77].

Vav proteins can be subjected to alternative transcriptional, epigenetic (methylation, microRNA), and posttranscriptional (arginine methylation, protein degradation) regulatory mechanisms [78,79,80,81,82,83,84,85,86,87,88]. Experimental evidence indicates that these extra regulatory steps do modulate the signaling output of these proteins. Thus, the transcriptional silencing of the *VAV1* gene in some clinical subtypes of T cell acute lymphoblastic leukemia has been shown to be essential to eliminate the tumor suppressor activity of Vav1 against the Notch1 pathway. As a result, the forced reexpression of Vav1 leads to impaired leukemogenesis in both T cell acute leukemia cell lines and patient-derived cells [37]. Conversely, the concurrent action of demethylation and transcriptional factors has been shown to be important for the ectopic expression of the *VAV1* gene in some types of nonhematopoietic tumors [85,87]. Similarly, the mouse *Vav3* gene has been shown to be transcriptionally activated by the aryl hydrocarbon receptor (Ahr) under normal physiological conditions [86,89]. Finally, the translocation of Vav1 to the nucleus depends on a nuclear localization signal present in the PH region, the potential binding of cytoplasmic retaining factors to most C-terminal Vav1 SH3 domain and, possibly, arginine methylation events [41,90]. Current data indicate that the list of regulatory layers affecting the biological activity of Vav proteins is larger than expected. For example, we have recently found that the anchoring of Vav1 in the plasma membrane can be enhanced by interactions with membrane-resident phosphatidylinositol-5 phosphates. This interaction is mediated by a composite motif formed by the Vav1 ZF and an adjacent polybasic tail (S.R-F. and X.R.B., manuscript submitted). We have also found that the acetylation of Vav1 on specific lysine residues leads to reduced levels of NFAT stimulation in T lymphocytes (S.R-F. and X.R.B., manuscript submitted). There is also evidence indicating that single nucleotide polymorphisms (SNP) in the *Vav1* gene might eventually affect the overall expression or the specific signaling output of the encoded protein [91,92]. For example, one of those SNPs is associated with the expression of a Vav1 variant harboring a single amino acid change (R63W) in the CH domain that is characterized by high levels of catalytic activity, lack of CH-dependent adaptor functions, and poor levels of expression in cells [92]. These SNPs correlate with immune alterations in several species [91,92,93,94]. SNPs for *VAV2* and *VAV3* genes have also been described associated with several diseases (for recent data, see [95,96,97,98,99]). At this point, however, no information is available regarding the effect that such SNPs cause in the biological activity of these proteins.

It is worth mentioning that most of the studies pertaining the regulation of this GEF family have used Vav1 as main experimental tool. It is likely that the rest of Vav family members will follow quite similar regulatory mechanisms given their common structure. However, we cannot exclude the possibility that each family member could have variations from this common regulatory framework. As a token, the binding of phosphatidylinositol-5 phosphates to Vav1 is not conserved in the other two family members (S.R-F. and X.R.B., manuscript submitted). Some of these regulatory layers are also cell type-specific given that, for example, both the lysine acetylation and the binding of phosphatidylinositol monophosphates contribute to the signaling output of Vav1 in lymphocytes but not in nonhematopoietic cells (S.R-F. and X.R.B., manuscript submitted).

## 5. Phylogenetic Evolution

**Figure 2:**
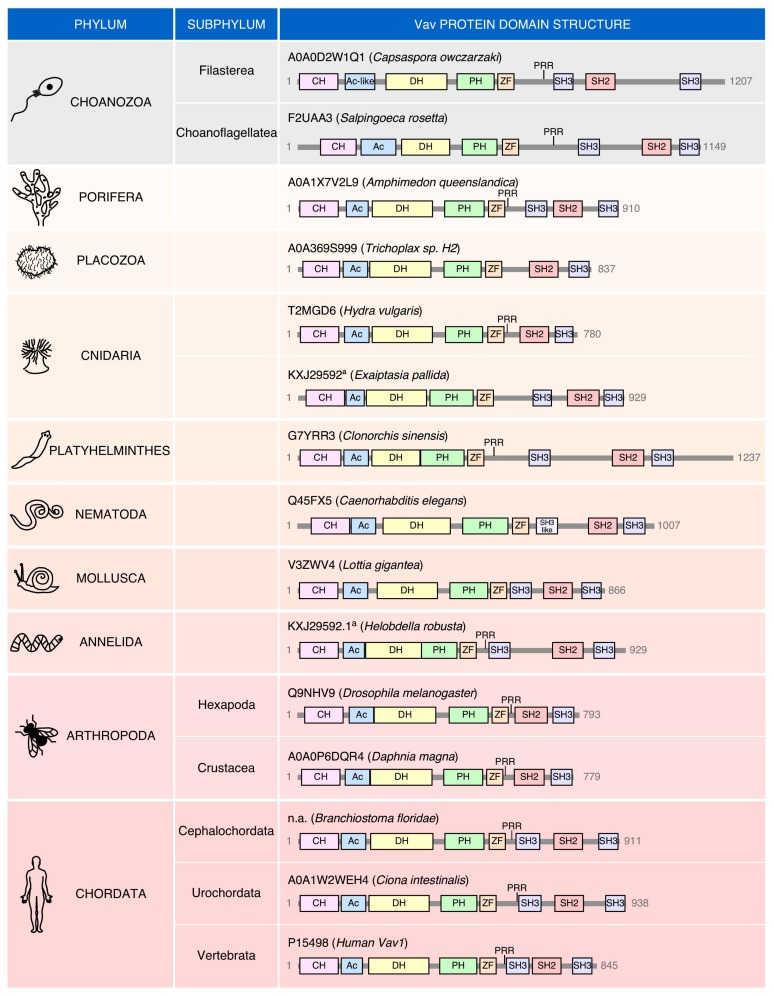
Evolution of the “body plan” of Vav family proteins. Depiction of the structure of Vav proteins in representative organisms of both the choanozoan (in gray) and metazoan (salmon-color gradient) phyla. UniProt accession codes are indicated for each protein.

No Vav family members have been found so far in the most basic animal unicellular organisms or in plants (Figure 1B). The most primitive family relatives are found in choanozoans (Figure 1B and Figure 2), a recently identified group of unicellular organisms considered to be the closest relatives to animal metazoans [100,101]. Choanozoans are believed to be the first cells in which PTKs have been ever developed (Figure 1B) [100,101]. By contrast, Rho GTPases and Rho GEFs arouse much earlier on in evolution (Figure 1B). This feature further underscores the close association of Vav proteins with PTK-regulated signaling pathways. Vav family members are present since then in all animal metazoan species that have been characterized by genome sequencing (Figure 1B and Figure 2). Interestingly, the analysis of all these proteins indicates that the “body plan” of the Vav family has been set very early on in evolution because, for example, the characteristic eight structural domains present in mammalian Vav proteins are already found in the choanozoan relatives (Figure 2). However, there are significant variations in terms of protein length, spatial arrangement of structural domains, and in the presence of specific domains among the species analyzed. For example, there are clades that contain Vav family proteins significantly larger (platyhelminths, choanozoans) and shorter (some cnidarians, arthropods) than those found in most species (Figure 2). These variations in length are due to the presence of larger intervening amino acid sequences located between the ZF-NSH3 (some choanozoans), the NSH3-SH2 (some choanozoans, platyhelminths) and the CSH3-Stop codon (filastereans and platyhelminths) regions (Figure 2). The shorter forms usually result from the elimination of the NSH3 domain (e.g., arthropods) (Figure 2). We surmise that these changes in protein length do not have to alter the usual regulatory mechanism of Vav proteins, given that the inhibitory CSH3 should still be able to establish interactions with the catalytic DH-PH-ZF cassette in the case of the longer family members (Figure 1A). In contrast to the structural variation in the C-terminal half of Vav proteins, the arrangement of the five most N-terminal domains (CH to ZF) remains relatively constant, showing only minor interdomain variations in all the species surveyed (Figure 2).

The localization of the PRR is also highly variable among species (Figure 2). In most cases (including vertebrates), these regions are in close proximity to the NSH3. However, in other cases, they can be found located farther away from the SH3 (e.g., choanozoans, porifera, and platyhelminths) (Figure 2). Some species also show the concurrent (placozoans) or independent loss of the PRR and NSH3 (cnidarians, nematodes, and mollusks in the case of the PRR; some cnidarians in the case of the NSH3) (Figure 2). Finally, in fewer cases, the missing PRR-NSH3 is substituted by a single, SH3-like region (nematodes) (Figure 2). These data suggest that the PRR might have intrinsic functions that are independent of the formation of a single structural unit with the NSH3 regions. They also indicate that none of those two regions must be essential for the activity of Vav proteins, an observation consistent with the mutagenesis experiments performed with the mammalian members of this GEF family [15,102]. The reason for this large variability in the organization of the C-terminal domains is unknown.

**Figure 3:**
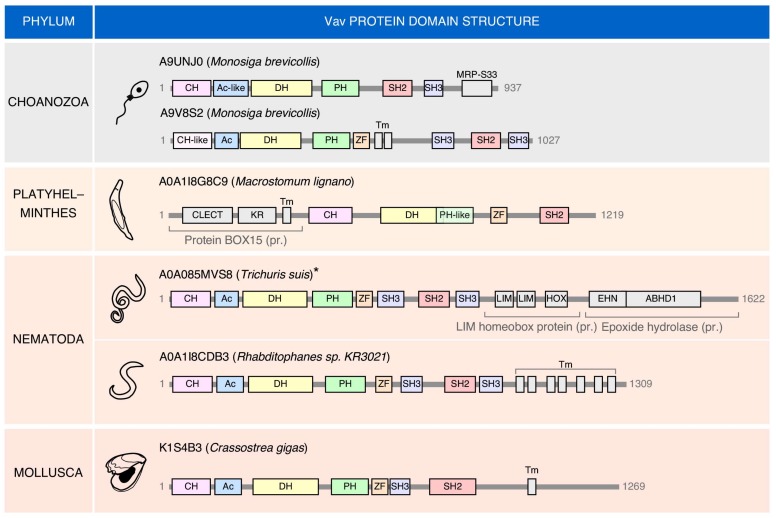
“Exotic” forms of Vav proteins found in invertebrate species.Depiction of the structure of Vav proteins in the indicated organisms (Uniprot accession codes are indicated for each protein). MRP-S33, mitochondrial ribosomal protein S33; Tm, transmembrane domain; CLECT, C-type lectin or carbohydrate-recognition domain; KR, kringle domain; LIM, zinc-binding domain present in Lin-11, Isl-1, and Mec-3; HOX, homeobox; EHN, epoxide hydrolase N-terminus; ABHD1, abhydrolase domain-containing 1. Please note that the relevance of the “exotic” version detected in *Trichuris suis* is still unclear since there is discrepancy in the overall structure of the locus in three independent animals of this species that have been sequenced. In the rest of species, they do seem to represent bona-fide loci.

Interestingly, there are cases in which “exotic” members of the Vav family can be found (Figure 3). These proteins share many of the typical structural domains present in the family. However, they also harbor atypical domains at the C-terminus (choanozoans, nematode, mollusks) or, to a lesser extent the N-terminus (the platyhelminth *Macrostomum lignano*). In this latter case, an atypical DH-PH domain is also found (Figure 3). The most frequent change is the presence of extra amino acid sequences containing putative transmembrane domains (Figure 3). As a result, of the incorporation of these new domains, the “exotic” Vav protein found in the nematode *Trichuris suis* represents the largest member of the entire family (1622 amino acids) found in all the species analyzed (Figure 3). These atypical versions can also lack some of the domains common to most Vav proteins such as the NSH3 (the choanozoan *Monosiga brevicollis*), the CSH3 (the mollusk *Crassostrea gigas*), and the two SH3 domains (the platyhelminth *Macrostomum lignano*) (Figure 3). These exotic versions seem to represent bona-fide proteins rather than sequencing artifacts in most cases. The functionality and roles of these evolutionary dead ends of the family remain unknown.

**Figure 4:**
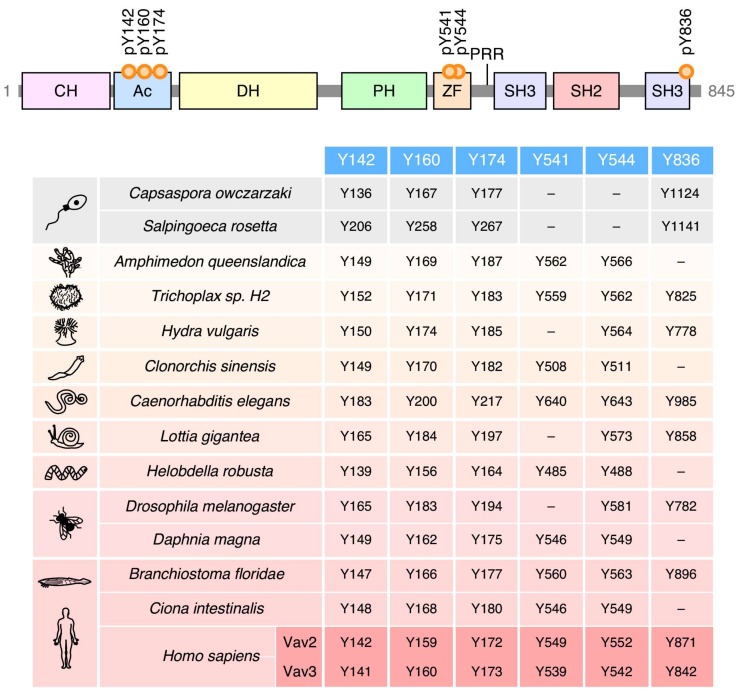
Conservation of the main phosphosites involved in Vav family regulation.For comparison, we include the structure and positions of the regulatory phosphosites of mouse Vav1 (top). Numbers (red boxes) indicate the position in the primary sequence of the indicated Vav family protein of the regulatory phosphosites that were previously identified in mammalian Vav1 (blue boxes).

Given the high level of structural conservation throughout the phylogenetic tree (Figure 2), it can be surmised that the main phosphotyrosine-dependent mechanism involved in the regulation of Vav proteins was already set at the level of choanozoans. Consistent with this idea, the four most important phosphorylation sites involved in the phosphorylation-mediated “opening” of Vav molecules are already present in species of this clade (Y^142^, Y^160^, Y^174^, Y^836^; according to the amino acid sequence of Vav1) (Figure 4). The Y^836^ site, however, was subsequently lost in cnidarians, platyhelminths, annelids, some arthropods (*Daphnia magna*), and some prochordates (*Ciona intestinalis*) (Figure 4). The less relevant Y^541^ and Y^544^ phosphosites seem to represent a latter acquisition, given that they are first found in porifera (Figure 4). Since then, the Y^541^ is lost in cnidarians, mollusks and some arthropods (*Drosophila melanogaster*) (Figure 4). There are no cases in which the Y^541^ and Y^836^ residues are concurrently lost in the same species (Figure 4), suggesting that these two phosphosites might contribute to the same regulatory step in the opening of the molecule.

**Figure 5:**
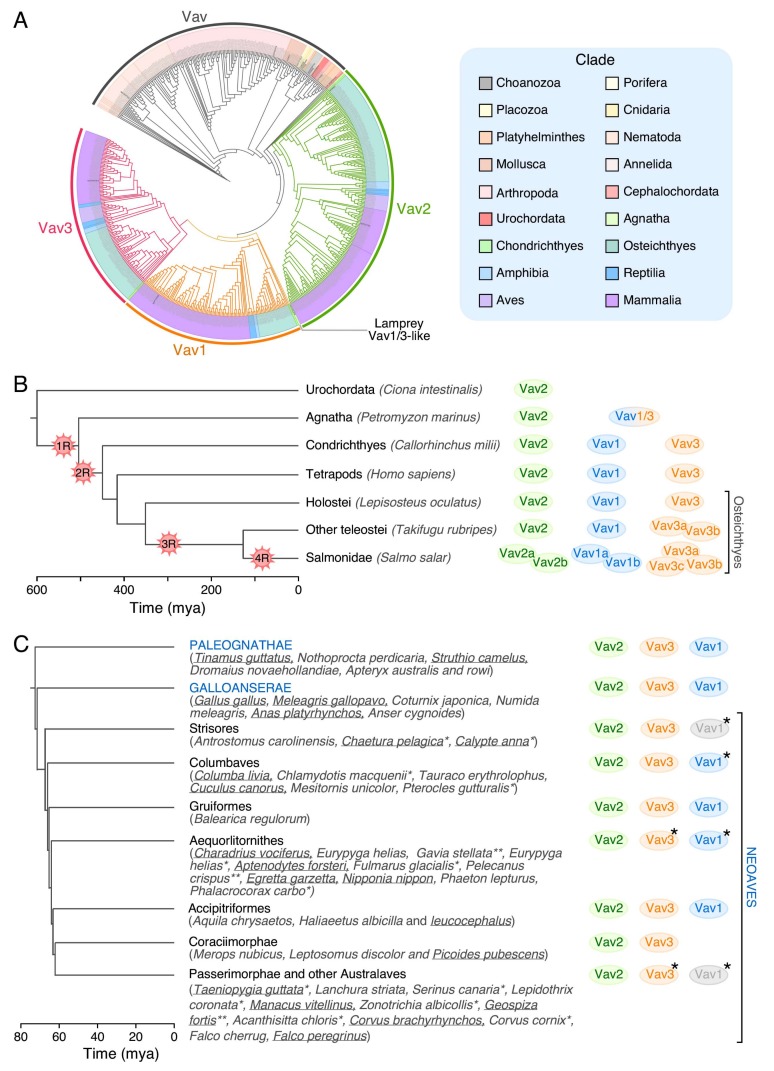
The gain and loss of Vav family members in vertebrates. (**A**) Phylogenetic tree of Vav family proteins (left). Proteins containing DH and SH2 domains were obtained from the UniProt database and the phylogenetic tree reconstructed using the maximum-likelihood method. Node colors indicate if the protein belongs to the single Vav family group (gray color) or the vertebrate Vav1 (orange color), Vav2 (green color) or Vav3 (purple color) branch. The lamprey (*Petromyzon marinus*) Vav1/3-like protein node has been highlighted in light brown. The color code used for each phylogenetic clade is shown on the light blue box (right). (**B**) Number of *Vav* family genes conserved in each vertebrate clade. 1R and 2R indicate the two rounds of whole-genome duplications that took place before the divergence of jawless and cartilaginous fish, respectively. 3R and 4R indicate the lineage-specific whole-genome duplication events associated with the origin of the ancestor of most teleost fish and salmonids, respectively. Time scale is indicated at the bottom (Mya; million years ago). (**C**) Variability in the number of Vav family proteins in avian clades. Species with either high-coverage or fully sequenced genomes are underlined. Cases associated with the loss of *Vav1* and/or *Vav3* genes in some species are indicated with asterisks. Proteins colored in gray indicate that the Vav family member has been lost in a high percentage of species of the indicated group. Avian phylogeny has been established as described elsewhere [105]. Time scale is indicated at the bottom.

Except for the choanoflagellate *Monosiga brevicollis*, which contains both a conventional and an “exotic” Vav family member (Figure 3), all the invertebrate and urochordate species that have been sequenced up to now contain a single Vav family gene (Figure 5A,B). In terms of sequence homology, these members can be regarded as analogs of the Vav2 protein present in vertebrates (Figure 5A,B). The first expansion in the number of family proteins is found in Agnatha, where we can detect a Vav2 analog and a second family member that shares structural features with the vertebrate Vav1 and Vav3 proteins (referred to here as Vav1/3) (Figure 5B). The three Vav proteins typically found in most vertebrate species are first found in chondrichthyes, probably as a consequence of a gene duplication event that affected the ancestral *Vav1/3* gene described above (Figure 5B). Interestingly, the origin of *Vav1* is associated with the point in evolution in which the adaptive immune system became fully developed [103]. It also matches the moment in which the ZF-polybasic tail cassette involved in the specific regulation of Vav1 by phosphatidylinositol monophosphates is first found in the phylogenetic tree (S.R-F. and X.R.B., manuscript submitted). A further increase in the number of *Vav* family genes is found in fish such as *Takifugu rubripes* (four genes) and *Salmo salar* (seven genes) (Figure 5B). This phenomenon is probably linked to the extensive whole-genome duplication events that took place at different moments of the evolution of teleost fish (Figure 5B). By contrast, the *Vav1* gene, the *Vav3* gene and, in some cases, both the *Vav1* and *Vav3* genes are lost during specific stages of the evolution of specific species of the neoavian clade (Figure 5C). It is likely that these gene losses are associated with the extensive genomic remodeling that took place at that time to promote the emergence of anatomical and metabolic features compatible with more efficient flying in those birds (e.g., increased muscle mass and thermogenic tissue, reduced bone structure) [104]. These gene losses took place quite rapidly in terms of evolutionary time (approx. 20 million years) [104]. Interestingly, all these genome remodeling events always spared the *Vav2* gene (Figure 5C).

## 6. Physiological Roles

The reason for the emergence of *Vav* genes in choanozoans is unknown. Although unicellular, the species of this phylum share some morphological features with animal metazoans and, in addition, can form benthonic multicellular colonies. They are also the first organisms in which PTKs are clearly present [101,106] (Figure 1B). It is likely therefore that Vav proteins might have evolved at this evolutionary stage to regulate PTK-dependent cytoskeletal processes that are required during the benthonic phase of these cells. Consistent with this idea, it has been shown that the *Vav* gene present in *Salpingoeca rosetta* is specifically expressed when this choanoflagellate shifts from the single cell to the colony stage [107].

Whereas the roles of these distant family relatives remain in the hypothetical realm, classical genetic analyses have shown that the single Vav family member present in the nematode *Caenorhabditis elegans* does play critical roles in the regulation of rhythmic contractile responses in the pharynx, ovaries, and intestine [48]. In addition, it is involved in the regulation of sleep-like behavioral quiescence, proper meiotic maturation, and neurogenic processes important for locomotion [108,109,110]. All these functions are PTK-dependent and Rho GTPase-mediated [48,108,109,110]. As it will be discussed below, some of these functions (neurogenic development, axon guidance, locomotor activity, regulation of contractility of some tissues) have been maintained in the case of the mammalian Vav2 and Vav3 proteins [46,111,112,113,114].

Genetic analyses indicate that the Vav protein present in *Drosophila melanogaster* participates in Rac1-mediated axon guidance processes that contribute to both larval viability and the locomotor activity of adult flies [115,116]. This protein is also involved in an epidermal growth factor receptor-triggered signaling pathway that contributes to the viability of the male stem cell niche [117,118]. The use of gain-of-function Vav mutants has additionally revealed that this GEF can participate in several Rac1-dependent developmental processes in this fly such as dorsal closure, myoblast fusion, the proper migration of caudal visceral mesodermal cells, and the formation of the tracheal system [72]. This protein also participates in the development of the eye using, in this case, both catalysis-dependent and independent mechanisms [119,120]. This is the first case, to our knowledge, in which a catalysis-independent function for Vav proteins has been found in invertebrate species.

No information is available regarding the role of Vav proteins in nonmammalian vertebrate species. In mammals, most of the genetic evidence regarding the role of Vav proteins has been gathered in mice. In the case of the most ancient member of the Vav family (Vav2), these data indicate that this family member is important for the generation of the peritoneal B_1_ cells, the stimulation of optimal humoral immune responses, and the proper relaxation of blood vessels induced by nitric oxide [46,121,122]. Due to this, the inactivation of the *Vav2* gene leads to defective B cell responses, hypertension, and hypertension-associated comorbidities in mice [46,121,122]. Additional known functions of Vav2 include the negative regulation of the cell surface expression and activity of the dopamine transporter in limbic dopaminergic neuron terminals. An important manifestation of this function is the diminished behavioral response of *Vav2*^−/−^ mice to the administration of cocaine [123]. Unpublished data indicate that Vav2 is also important for the regulation of both insulin growth factor 1 and insulin signaling in skeletal muscle. In agreement with this, mice bearing a mutant Vav2 protein with decreased catalytic activity exhibit reduced muscle mass and insulin responsiveness that eventually favor the development of a metabolic syndrome-like condition. Conversely, mice expressing a catalytically hyperactive Vav2 protein develop muscle hypertrophy, increased insulin responsiveness, and protection against high fat diet-triggered dysfunctions and obesity (S.R-F and X.R.B., manuscript submitted).

Vav3 is involved in a variety of cerebellar functions such as Purkinje cell dendritogenesis, the survival and migration of granule cells, and the formation of the cerebellar intercrural fissure during the perinatal period. This leads to motor coordination and gaiting defects in early postnatal periods when Vav3 is genetically inactivated in mice [112]. Vav3 also controls the migration of axons of GABAergic neurons from the caudal to the rostral region of the ventrolateral medulla [114], a brainstem center that controls sympathetic, respiratory, heart, cardiovascular, and renal activities [124]. When Vav3 is absent, this GABAergic wiring is not properly established, leading to the loss of the tonic inhibition exerted by the ventral over the rostral area of the ventrolateral medulla, and the development of multiple sympathetic-dependent physiological defects in mice [111,114,125,126]. Further functions of Vav3 inside the nervous system include the blockage of the differentiation of oligodendrocyte precursors and the promotion of myelination processes during both development and injury recovery [127]. Outside the central nervous system, Vav3 has been linked to the regulation of the differentiation of progenitor cells during the development of the mouse retina, the permeability of endothelial cells, the maturation of osteoclasts, and the regulation of antifungal innate immunity [95,128,129,130].

Consistent with an evolutionary origin linked to the development of the adaptive immune system, the mammalian Vav1 protein is mainly involved in the development, positive selection, negative selection, and effector functions of T lymphocytes (for reviews, see [9,131]). More recent results indicate that Vav1 is also important for the homeostatic production of regulatory T cells [132]. Most of these functions are believed to be dependent on the catalytic activity of this protein. However, the use of knock-in mice expressing Vav1^R63W^, a natural variant that harbors a CH domain that lacks adaptor functions [92] (see Section 4), has revealed that the adaptor functions of this protein can also contribute to the intrathymic negative selection of thymocytes and the production of regulatory T cells [93,94]. The most recently reported function for Vav1 is the participation in a CD226-activated pathway that contributes to the amplification of T cell receptor responses and interleukin 17 production in helper T cells [133]. The important role of Vav1 in T cell signaling has also been observed in rats bearing a specific *Vav1* SNP [93,134]. Vav1 plays minor functions in a large variety of other hematopoietic lineages as well (for a review, see [9]).

Analyses using compound *Vav* family knockout mice have revealed that these proteins are also involved in axon guidance, dendritic spine formation, synaptic plasticity, enterocytic differentiation, intestinal epithelial barrier integrity, and neoangiogenesis [113,135,136,137]. It is not known at present, however, whether these functions require the redundant participation of several family members or just single Vav proteins.

## 7. Final Remarks

Despite the progress made, there are still pending questions regarding the regulation, conformational dynamics, functions, and potential therapeutic value of these proteins. Regarding the former issue, it is important to note that most studies have been done using Vav1 as main working model. This is probably not a problem in the case of the phosphorylation-dependent mechanism involved in the regulation of Vav proteins, since experimental evidence indicates that it is fully conserved in Vav2, Vav3, and the family relatives present in nematode and flies [4,13,48,72]. However, it is plausible that additional regulatory layers could affect the activity of these proteins in a family member- and/or tissue-specific manner. The recent observation that phosphatidylinositol monophosphates specifically affect the lymphocyte functions of Vav1 but not of the other family members supports this possibility. Furthermore, recent proteomics experiments have revealed that Vav proteins can undergo a large variety of posttranslational modifications. Current evidence suggests that some of them could be in fact functionally relevant. For example, we have recently found that the acetylation state of Vav1 on specific lysine residues does influence the levels of stimulation of the downstream NFAT pathway (S.R-F. and X.R.B., manuscript submitted). There is also evidence indicating that Vav proteins can be regulated by transcriptional mechanisms, protein-protein interactions, and microRNAs [4]. However, many of these potential regulatory layers await full characterization. Further studies are clearly needed to clarify the relevance, specific role, and level of phylogenetic conservation of these new potential regulatory layers.

As summarized in this review, we now have a rather good idea regarding the main interdomain interactions that maintain the “closed” conformation of Vav proteins in the nonphosphorylated state. In addition, crystallographic studies have revealed the specific amino acid residues that mediate the interaction of the CH-Ac region and the catalytic cassette in the inactive state of Vav1 protein [10] (Figure 1A). However, we still do not know the specific residues that mediate the inhibitory interaction of the Vav CSH3 domain with the rest of the molecule. We also lack information regarding the conformational transitions that Vav proteins undergo during the phosphorylation-mediated activation step, the signaling relevance of each of those conformational states, and the final spatial arrangement that all the structural domains of Vav proteins adopt in the context of the fully active conformation. These structural issues raise additional functional questions such as, for example, whether Vav proteins respond to extracellular stimuli using digital or analog signaling mechanisms, whether there are pools of phosphorylated Vav proteins endowed with different downstream signaling capacities, and whether the stimulation of the catalytic and adaptor functions of these proteins are always synchronized along the stimulation cycle of cells. All these issues can be approached in the near future using a combination of electron microscopy-based structural determination methods and signaling techniques.

We have learnt a lot of information regarding the biological functions of Vav family proteins during these last years. However, it is likely that we are far from understanding the full portfolio of functions that are executed by them. We should not forget either that Vav proteins, as probably many other multidomain Rho GEFs, might play roles outside the long-held signaling archetype for Rho GTPases [3]. The serendipitous discovery of the implication of Vav1 in the negative regulation of the Notch1 pathway is a good example of the signaling “black matter” that we still face in this particular area [37,38,39]. Given the noncanonical nature of these hypothetical functions, it is likely that they could only be unraveled upon the detection of unexpected phenotypes in genetically modified animal models.

We also must make further inroads in the understanding of the level of functional redundancy that exists among the different members of the family. Current data indicate that these proteins elicit common, synergistic, and specific pathways in cells [4,40]. However, it is still unclear whether this is due to different catalytic specificity, affinities for upstream PTKs, the spectra of protein partners, the subcellular distribution and/or the levels of expression of each family member. To solve this issue, we will probably have to focus on the analysis of endogenous proteins rather than in the use of ectopic expression methods. This strategy can be facilitated, for example, if we tag them with specific epitopes using CRISPR-Cas9-based gene editing techniques in cells. The side by side comparison of single and compound Vav family knockout mice can provide further information to answer this lingering question. Finally, we also have a long way to go to fully understand the function of the Vav family members present in other model organisms.

We have not dealt in this review with the potential therapeutic value of Vav proteins. Current evidence from Vav family knockout mice suggests that the inhibition of these proteins could be of interest in cancer, immune system-related diseases, and other pathologies [4]. This idea has been further reinforced by the recent identification of *VAV1* gene mutations in human tumors [9,38,73,74,75,76,77]. There are, however, pending questions in this area that have to be addressed before embarking on the development of Vav family inhibitors: **(i)** The potential side effects derived from the systemic inactivation of these proteins (see above, Section 6) and how to surmount them. **(ii)** The level of contribution of the catalysis-dependent and independent pathways of these proteins to the pathogenic programs of those diseases (see above, Section 3). **(iii)** The tumor suppressor-like functions regulated by some of these proteins [37]. **(iv)** The actual feasibility of developing high affinity inhibitors for these proteins, a problem that has been difficult to solve in the case of other Rho GEFs [3]. Fortunately, the idiosyncratic DH-PH-ZF catalytic cassette present in Vav proteins might offer additional drug pockets to circumvent this potential problem.

Although experimentally challenging and time-consuming, most of these pending issues can be tackled with using already available techniques.
